# Neural network dose prediction for rectal spacer stratification in dose‐escalated prostate radiotherapy

**DOI:** 10.1002/mp.15575

**Published:** 2022-03-11

**Authors:** Christopher Thomas, Isabel Dregely, Ilkay Oksuz, Teresa Guerrero Urbano, Tony Greener, Andrew P. King, Sally F. Barrington

**Affiliations:** ^1^ School of Biomedical Engineering & Imaging Sciences King's College London London UK; ^2^ Medical Physics Department Guy's and St. Thomas’ Hospital NHS Foundation Trust London UK; ^3^ Computer Science Department UAS Technikum Wien Vienna Austria; ^4^ Computer Engineering Department Istanbul Technical University Istanbul Turkey; ^5^ Clinical Oncology Department Guy's and St. Thomas’ Hospital NHS Foundation Trust London UK; ^6^ Clinical Academic Group King's College London London UK; ^7^ King's College London and Guy's and St. Thomas’ PET Centre, School of Biomedical Engineering and Imaging Sciences King's Health Partners London UK

**Keywords:** dose prediction, prostate radiotherapy, rectal spacer, rectal toxicity, stratify

## Abstract

**Purpose:**

To develop a knowledge‐based decision‐support system capable of stratifying patients for rectal spacer (RS) insertion based on neural network predicted rectal dose, reducing the need for time‐ and resource‐intensive radiotherapy (RT) planning.

**Methods:**

Forty‐four patients treated for prostate cancer were enrolled into a clinical trial (NCT03238170). Dose‐escalated prostate RT plans were manually created for 30 patients with simulated boost volumes using a conventional treatment planning system (TPS) and used to train a hierarchically dense 3D convolutional neural network to rapidly predict RT dose distributions. The network was used to predict rectal doses for 14 unseen test patients, with associated toxicity risks calculated according to published data. All metrics obtained using the network were compared to conventionally planned values.

**Results:**

The neural network stratified patients with an accuracy of 100% based on optimal rectal dose–volume histogram constraints and 78.6% based on mandatory constraints. The network predicted dose‐derived grade 2 rectal bleeding risk within 95% confidence limits of ‐1.9% to +1.7% of conventional risk estimates (risk range 3.5%–9.9%) and late grade 2 fecal incontinence risk within ‐0.8% to +1.5% (risk range 2.3%–5.7%). Prediction of high‐resolution 3D dose distributions took 0.7 s.

**Conclusions:**

The feasibility of using a neural network to provide rapid decision support for RS insertion prior to RT has been demonstrated, and the potential for time and resource savings highlighted. Directly after target and healthy tissue delineation, the network is able to (i) risk stratify most patients with a high degree of accuracy to prioritize which patients would likely derive greatest benefit from RS insertion and (ii) identify patients close to the stratification threshold who would require conventional planning.

## 1 INTRODUCTION

Prostate cancer is the most common cancer in men in the United Kingdom (UK), with over 48 000 cases diagnosed and 11 000 deaths per year.^1^ Thirty percent of patients receive radiotherapy (RT) as part of their treatment.^1^ Despite the latest developments in image‐guided and highly conformal delivery techniques, RT to the prostate gland may leave patients with rectal toxicities which can severely impact quality of life. To combat rectal toxicity, rectal spacing devices can be surgically inserted through the perineum to lie between the prostate and the anterior rectal wall. This strategy has been reported to reduce dose to the rectum with fewer acute toxicities.^2^

The use of rectal spacers (RS) has been approved by the UK National Institute for Clinical Excellence (NICE),^2^ yet the cost of spacer and surgical insertion is not routinely funded by the UK National Health Service (NHS). Hence, with limited resources a decision‐support system that can accurately prioritize high‐risk patients most likely to derive benefit from RS insertion is required.^3^ Increased radiation dose to the rectum is associated with increased acute and late rectal toxicity,^4^ so prediction of rectal dose is important for estimating toxicity risk in a decision‐support system. Prediction of rectal dose and associated toxicity could play an especially valuable role in dose escalation treatment strategies, currently being explored in phase III trials,^5^, ^6^, ^7^ where rectal doses are likely to be higher. The conventional method for predicting rectal dose from RT is to acquire a treatment planning computed tomography (CT) scan, delineate target volumes and healthy organs, and generate a treatment plan. However, existing methods for RT treatment planning are resource and time intensive, user dependent, and subject to clinical workload pressures. Within recent years, neural networks (NNs) have been developed^8^, ^9^, ^10^, ^11^, ^12^, ^13^ to predict dose distributions for unseen patients, often in a matter of seconds, based on internal anatomy and knowledge of previous RT distributions. This works sets out to develop a rapid and streamlined process to risk‐stratify patients in terms of rectal toxicity directly after anatomical delineation, thus eliminating the need for risk estimation via time‐ and resource‐intensive treatment planning.

The aims of this proof of principle study were to design and train a NN to (i) rapidly predict rectal dose distributions for unseen patients planned for dose‐escalated prostate RT, and (ii) stratify high‐risk patients for RS insertion.

## 2 MATERIALS AND METHODS

### 2.1 Treatment planning

**FIGURE 1:**
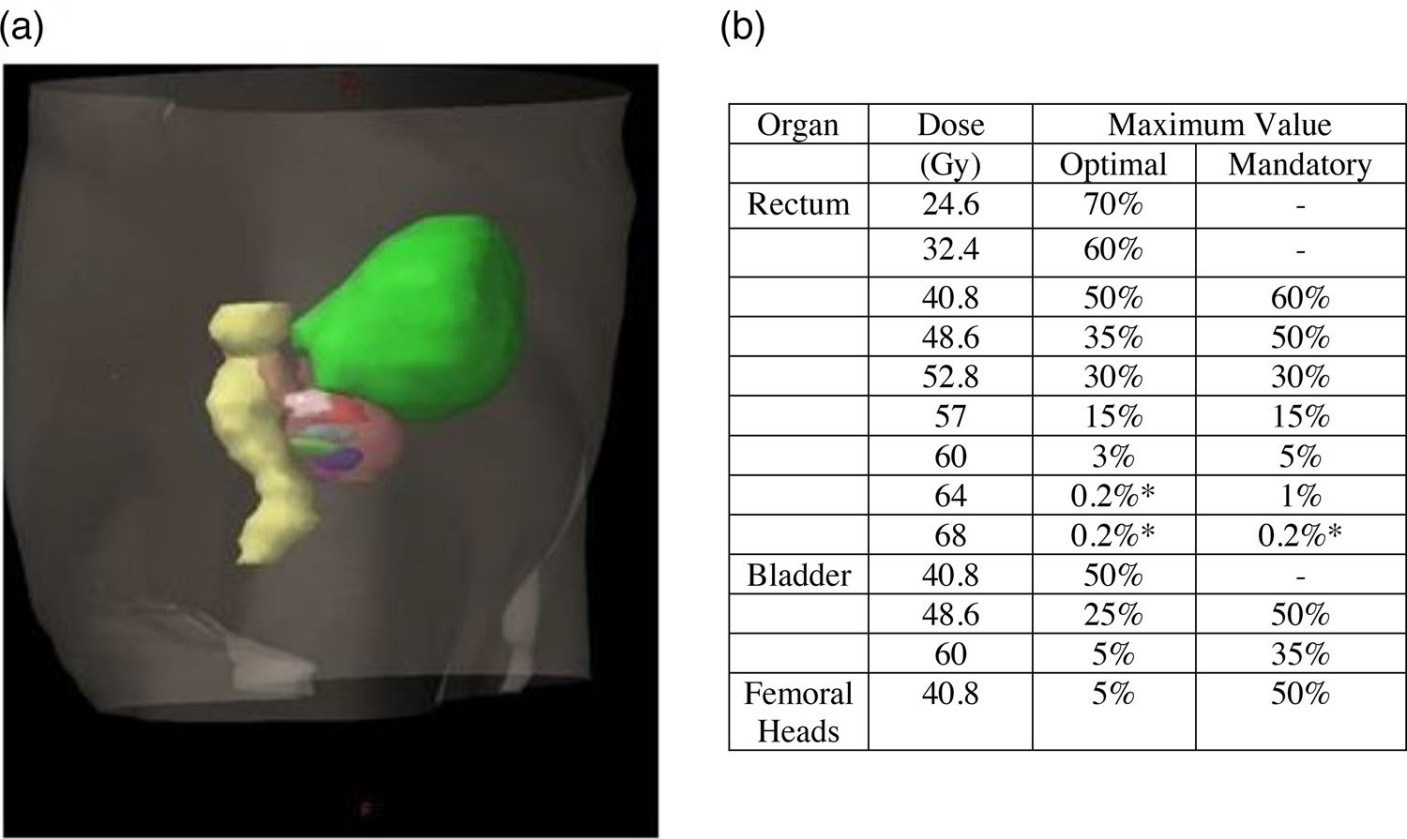
(a) Three‐dimensional projection of simulated boost regions planning target volume (PTV)68‐A to PTV68‐F shown as colored regions within clinical target volume (CTV)60 volume (pink). Rectum (yellow) and bladder (green) are also shown. (Colors shown in online version only.) (b) Organ at risk (OAR) dose tolerances table modified from Onjukka et al.^14^ *0.2% tolerance was chosen for practical treatment planning reasons to replace 0% tolerance

Forty‐four patients with histologically proven prostate cancer were enrolled at Guy's and St. Thomas’ NHS Foundation Trust into a local Research and Development and ethical review board approved clinical study (NCT03238170) registered on clinicaltrials.gov. All patients gave written informed consent and were treated with standard of care RT. For this study, clinical target volumes (CTVs) delineated according to institutional protocol were used to grow planning target volumes (PTVs) according to the dose escalation pilot study,^14^ which acted as a precursor to the national PIVOTALBoost phase III trial.^7^ PTV60 is CTV60 (prostate gland) with 5 mm geometric expansion, and PTV53 is CTV53 (prostate and seminal vesicles) with 9 mm margin. Thirty consecutive patients were selected for training. For each of these patients, six simulated lesions were created on the planning CT, to represent dominant intra‐prostatic lesions (DILs) (Figure 1a). All simulated DILs were positioned in the peripheral zone where the majority of foci arise clinically^15^, ^16^ and where posteriorly located lesions result in the highest rectal toxicity risk.^17^ For each of the simulated boost DILs, a 3 mm geometric margin was added to create each CTV68, then cropped to the edge of the prostate. A geometric 2 mm PTV margin was added creating six PTVs labeled PTV68‐A to PTV68‐F. Boost DIL regions were intentionally created with volumes similar to those in the literature^6,18^: median (range) for boost DIL, CTV68 and PTV68 volumes were 3.1 cc (0.3–10.8 cc), 6.8 cc (1.1–19.6 cc), and 13.0 cc (3.0–31.7 cc). For the remaining 14 patients (test cohort), clinically significant boost regions were delineated by a trained clinical oncologist according to PIRADSv2 from multi‐parametric magnetic resonance imaging sequences and histological reports. For all patients, organ at risk (OAR) delineation comprised the bladder, rectum, and left and right femoral heads. The full trans‐axial extent of the rectum was outlined from anal verge to recto‐sigmoid junction.

One hundred and ninety‐four RapidArc™ treatment plans were created, one for each simulated boost treatment in the training dataset and one for each patient in the test dataset, in Eclipse v13.6 treatment planning system (TPS) (Varian Medical Systems, Palo Alto, CA, USA) by an experienced treatment planner (12 years’ experience) using a standardized planning template to initialize the optimization stage. PTV and OAR dose constraints were based on those detailed in the PIVOTALBoost pilot study.^14^ OAR planning constraints are shown in Figure 1b. The single‐planner, template‐guided planning strategy, using well‐defined tolerances from a published clinical study allowed, as far as practicable, to develop a standardized dataset.

Treatment plans have 95% of PTV60 covered by 60 Gy, greater than 95% of PTV53 covered by 53 Gy, and median dose of 68 ± 0.3 Gy to PTV68 (the DIL PTV). PTV dose coverage was optimized to cover entire PTVs rather than compromising PTVs for rectal sparing. In this scenario, the planning aim was to reduce rectal dose as far as possible while maintaining maximal tumor control probability (TCP) by complying with the dose prescription specified above.

### 2.2 Data preparation for NN input

For each patient, PTV68A‐F, PTV60, PTV53, rectum, and bladder were converted to binary masks using TomoMask v1.4.1 (www.tomomask.com) and loaded into a Python environment with the corresponding dose distribution created using the TPS. Dose arrays were resampled using a third‐order spline function to 512 × 512, 0.98 mm in‐plane resolution corresponding to the binary structure masks and normalized using the maximum dose within all training datasets. In order to focus dose prediction on the area of interest, training and testing were cropped to 3D volumes (array size 128 × 128 × 64) centered left–right according to PTV53 geometric center of mass, craniocaudally according to the rectal center of mass and anterior–posteriorly such that the entire rectum was sampled. The entire rectal volume was encompassed by the 3D array for all patient plans.

### 2.3 Network architecture and training

A five‐level, 3D Hierarchically Densely Connected U‐Net^8^ (HD U‐net) was constructed with (3 × 3 × 3) convolutions reducing the feature size from 128 × 128 × 64 pixels to 8 × 8 × 4 pixels. Rectified linear unit activation functions were performed after each convolution in the contracting and expansive paths, and a linear activation function utilized for the final (1 × 1 × 1) convolution. The Adam optimizer was used, with learning rate 10^−4^, and mean squared error loss function was minimized.

Initial network hyper‐parameter tuning of learning rate, kernel size, number of network levels, and epochs was performed using 20 of the 30 training patients in a leave one‐out cross‐validation approach (validation cohort), with 19/20 patients comprising the training set for each fold. Leave one‐out cross‐validation was chosen as it is more informative than validation with larger folds (e.g., fivefold). The process is however more time consuming, hence the decision to perform validation and tuning with 20 patients from the full 30‐patient training cohort. The sampled 3D volumes consisted of 128 × 128 × 64 pixels, therefore each training fold had input dimensions [114, 5, 128, 128, 64] where the first element represented six boost plans for the 19 patients and the second element the five binary structure masks: PTV68, PTV60, PTV53, rectum, and bladder. Loss was calculated against the manually planned dose distributions [114, 1, 128, 128, 64] for each training treatment plan. The dose distributions were then predicted for the six treatment plans for the left‐out patient, with output dose predictions rescaled to dose using the maximum dose from the validation cohort.

The tuned network parameters were utilized for training on all 30 training patients, to generate a model ready for testing on the test cohort of unseen 14 patients with clinically derived lesions.

### 2.4 Rectal dose and toxicity risk modeling

Predicted rectal dose–volume histograms (DVHs) were extracted from the predicted dose arrays and compared with manually planned DVHs. Rectal DVHs from manual plans and predicted plans were imported into the commercially available software BioSuite.^19^ Lyman–Kutcher–Burman (LKB) model parameters (TD_50_ = 97.7 Gy, *m* = 0.27, *n* = 0.085, *α*/*β* = 3 Gy) were applied to predict the risk of grade 2 (G2) late rectal bleeding (LRB), and the risk of late fecal incontinence (LFI) (TD_50_ = 105 Gy, *m* = 0.43, *n* = 1, *α*/*β* = 3 Gy) as adopted by Onjukka et al.^14^ In all cases, DVHs were corrected for 2 Gy per fraction equivalence using *α*/*β* of 3 Gy.

To assess the accuracy of predicted 3D dose distributions, all isodoses fully encompassed within the predicted 128 × 128 × 64 voxel volumes were evaluated against planned isodoses using the dice similarity coefficient (DSC = 2(*A* ∩ *B*)/(*A* + *B*), where *A* and *B* represent the voxels within NN predicted and manually planned isodoses contours, and ∩ is the intersection).

To put our prediction results into clinical perspective, in a retrospective investigation, prostate and rectal volumes, as well as rectal doses were collated for a separate cohort of 100 consecutive patients receiving RT for prostate cancer at our institution, with the rectal doses converted to toxicity risk using LKB parameters above.

### 2.5 RS stratification

Four methods for dose‐derived RS stratification were simulated for this work: treatment plans where (i) optimal rectal DVH constraints were exceeded, (ii) mandatory rectal DVH constraints were exceeded, (iii) risk of G2 LRB, or (iv) risk of LFI was higher than specified thresholds. Network performance was evaluated for multiple risk thresholds to determine accuracy at differing stratification levels.

## 3 RESULTS

All manually generated plans met PTV objectives. Optimal and mandatory rectal constraints were exceeded for 161 and 57 of the 194 treatment plans, respectively, primarily at the higher doses as demonstrated for validation cohort and test cohort in the Supporting Information (Tables S1 and S2). This was anticipated as the treatment planning process aimed to maintain TCP by preserving PTV dose coverage. Median LKB predicted risk of G2 LRB toxicity in the study cohort was 7.4% (range 3.3%–10.4%) and median LKB predicted risk of LFI was 3.9% (range 2.3%–7.1%) (Figures S1 and S2). All manually generated treatment plans met femoral head and bladder tolerances.

### 3.1 Dose prediction

Training of the final network for 200 epochs took 5.4 h on a 12 Gb Titan Xp GPU. Deployment of the trained model on each 128 × 128 × 64 3D volume in the test cohort took less than 0.7 s.

**FIGURE 2:**
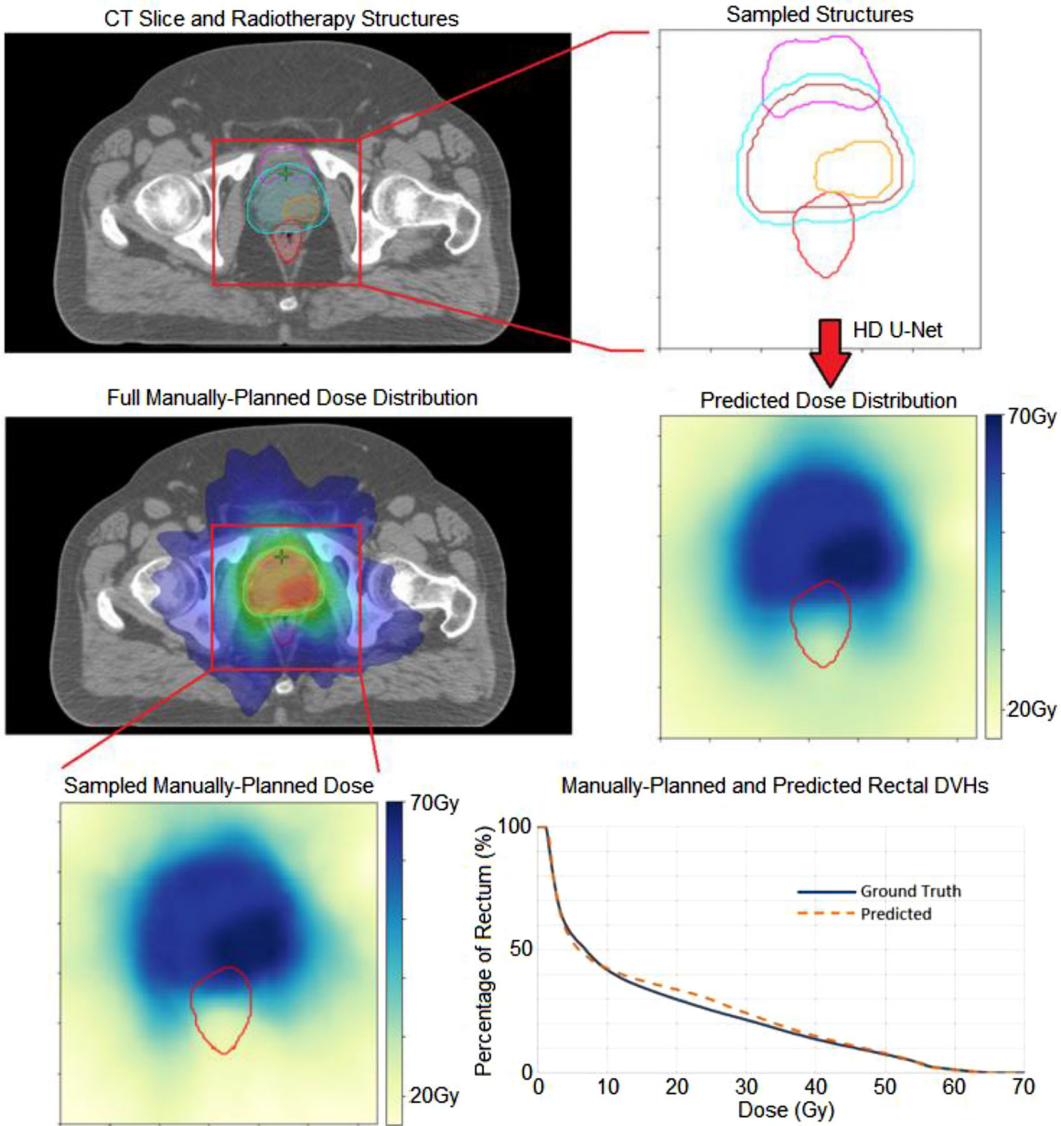
Dose prediction for patient 1 in test dataset. Structures shown are bladder (magenta), rectum (red), planning target volume (PTV)53 (cyan), PTV60 (brown), and PTV68 (orange). The predicted dose–volume histogram (DVH) follows the ground truth as visualized in the bottom right graph (Colors shown in online version only.)

Figure 2 shows the dose prediction and evaluation process for one representative patient plan.

Unless explicit reference is made to validation cohort or study cohort, the results quoted below refer to model testing on the unseen test cohort of patients with clinically derived boost regions.

Dose prediction using the network was highly accurate for PTVs (Table S4), with prediction of median dose 0.1 Gy higher than manual plans on average, with a low standard deviation (SD) of 0.3 Gy. PTV maximum (Dmax) and mean (Dmean) doses were also accurately reported (Table S5), with average absolute dose differences less than 2.1% (1.3 Gy) and 0.5% (0.3 Gy), respectively.

**FIGURE 3:**
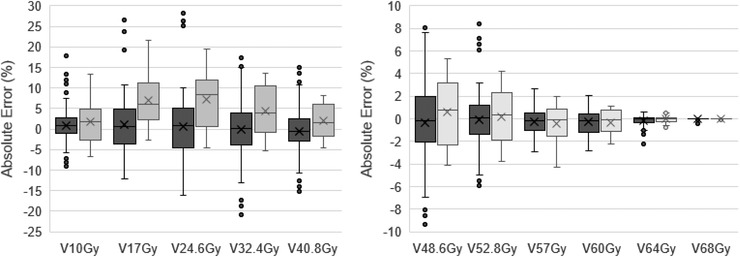
Prediction error from manually planned rectal dose–volume histogram (DVH) parameters for validation cohort (dark gray), and test cohort (light gray). Boxes represent interquartile range (IQR), within which are shown the median (line) and mean (cross). Top whisker represents the largest value within 1.5 times the IQR, above the box. Lowest whisker represents the smallest value within 1.5 times the IQR, below the box

Rectal dose prediction resulted in low bias for rectal DVH parameters above 40 Gy as shown in Figure 3, with mean dose prediction error less than 2%, and below 7.2% for the entire dose range.

Average absolute dose differences for rectal Dmax and Dmean were 1.4% (0.8 Gy) and 3.9% (2.3 Gy), respectively (Table S5). Results for 3D dose prediction accuracy are included in the Supporting Information, where average DSC for predicted isodoses up to and including the prescription dose was 0.94 (range 0.90–0.96), with mean SD of 0.011 (Figure S3).

When comparing test results with those at the validation stage (Figure 3) it can be seen that the positive bias was higher for the former but ranges were similar. An increase in predictive accuracy is witnessed at higher and lower doses for both test and validation cohorts.

**FIGURE 4:**
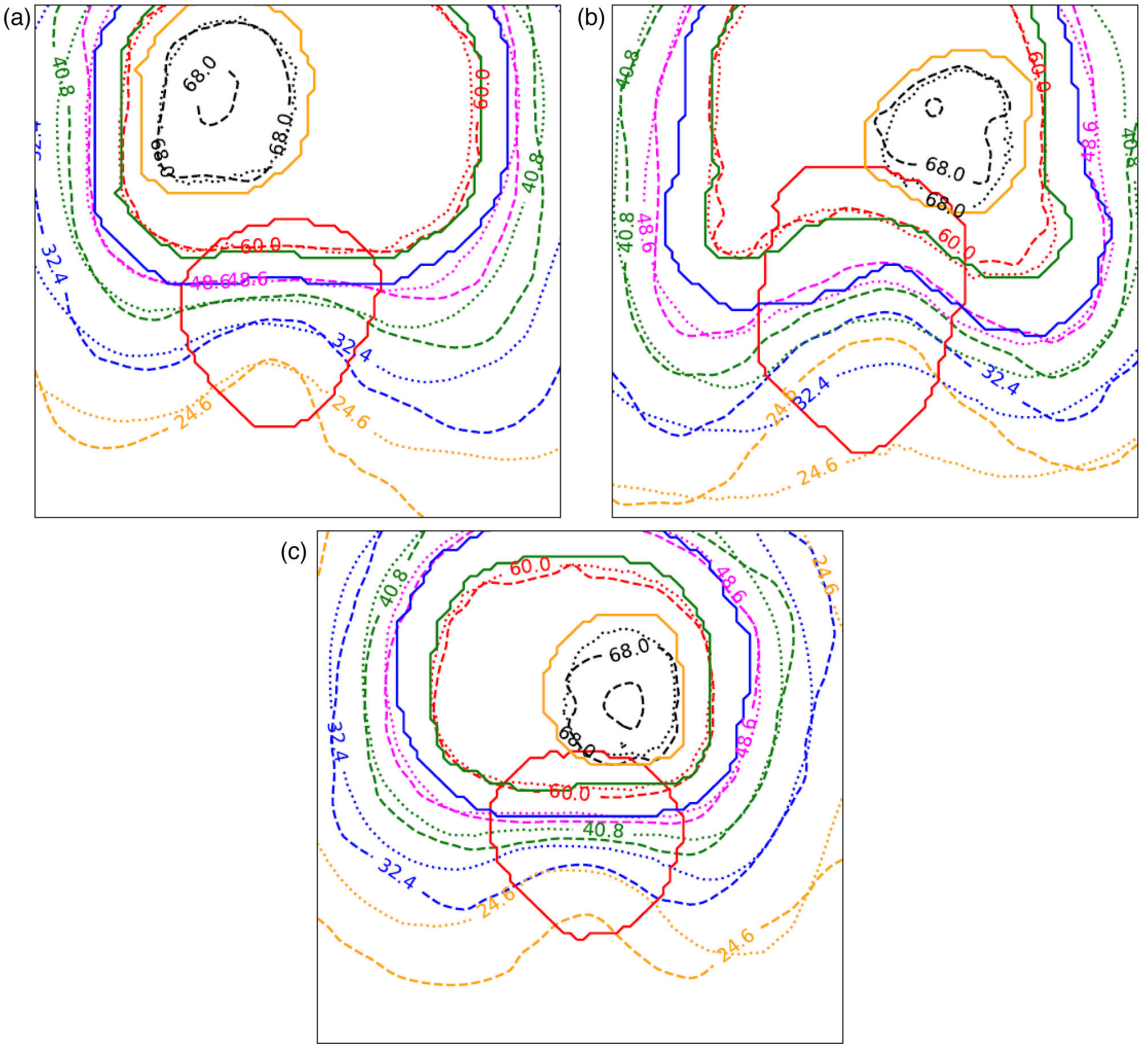
Axial slice through planning target volumes PTVs and rectum for (a) patient 14, (b) patient 4, and (c) patient 7. Solid lines indicate anatomical contours: rectum (red), PTV53 (blue), PTV60 (green), and PTV68 (orange). Dashed and dotted lines indicate manually planned and predicted isodoses, respectively. 24.6 Gy (orange), 32.4 Gy (blue), 40.8 Gy (green), 48.6 Gy (magenta), 60 Gy (red), and 68 Gy (black) (Colors shown in online version only.)

Figure 4 shows representative examples of dose prediction using the network compared with manually planned dose, representing three distinctly different outcomes. All examples show the concave distribution of isodoses within the rectum, indicating that the manually planned dose distribution has been optimized for rectal sparing and that the network also predicts this effect. The figure shows situations where rectal dose sparing is (a) predicted accurately, (b) underestimated, and (c) overestimated. In each example, the isodose agreement improves around the PTVs, as the influence of the rectum is reduced.

### 3.2 Risk prediction

**FIGURE 5:**
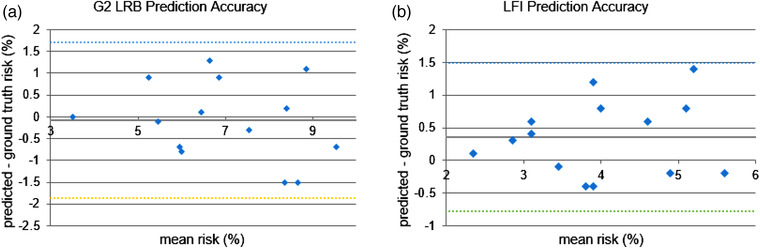
Bland–Altman plots of neural network (NN) risk prediction in the test cohort for (a) grade 2 (G2) late rectal bleeding (LRB) and (b) late fecal incontinence (LFI), where mean accuracy is shown as thick solid line and upper and lower 95% confidence limits as dotted lines

The Bland–Altman plots in Figure 5a,b show good agreement between toxicity estimations from manual plans (ground truth) and from estimations predicted by the network. Mean error for G2 LRB is ‐0.1% with +1.7% and ‐1.9% upper and lower 95% confidence limits, respectively. For LFI, mean error is 0.4%, with +1.5 and ‐0.8% upper and lower 95% confidence limits.

Rectal volumes in the study cohort (mean (*M*) = 69.3 cc, SD = 19.7 cc) were not significantly different (*p* = 0.903, *T*‐test) from the 100 clinically treated patients (*M* = 69.7 cc, SD = 20.6 cc). Prostate volumes within the study (*M* = 42.3 cc, SD = 15.7 cc) were comparable (*p* = 0.016) to the patient data (*M* = 51.3 cc, SD = 22.0 cc). Toxicity risk for the last 100 clinically treated patients had a similar distribution to the study population of 44 patients (Figures S1 and S2), with slightly higher mean LFI risk (4.8% compared with 4.2%) likely on account of larger PTV margins but slightly lower mean LRB risk (6.8% compared with 7.5%) probably due to absence of a dose escalation region. The network predicted late G2 LRB toxicity risk with a SD (0.9%) for prediction error lower than the SD (1.3%) of manually planned clinical risk (*p* = 0.114, using statistical *F*‐test). Prediction of LFI risk has significantly lower SD, with 0.6 compared to 1.2 (*p* = 0.005).

### 3.3 Dose‐based RS stratification

In the test cohort, five patient plans met optimal rectal DVH planning constraints and nine plans exceeded the constraints. In a scenario where RS insertion would be offered to patients considered high risk (whose treatment plan failed optimal rectal dose constraints), the prediction network resulted in a stratification accuracy of 100% into high‐ and low‐risk groups (Table 1a), correctly predicting those that passed the constraints (five plans) and those that failed (nine plans). Based on mandatory constraints, stratification accuracy was 78.6% (Table 1b), whereby nine out of the 10 plans meeting mandatory rectal DVH constraints were correctly identified and two out of four exceeding mandatory constraints were correctly stratified.

**TABLE 1:** Number of manual treatment plans and network predicted treatment plans meeting or exceeding (a) optimal and (b) mandatory rectal dose–volume histogram (DVH) constraints

	Manual plans	Network prediction	
(a)			
Within optimal DVH tolerances	5 (35.7%)	True negative	5 (100.0%)
		False negative	0 (0%)
Exceeding optimal DVH tolerances	9 (64.3%)	True positive	9 (100.0%)
		False positive	0 (0%)
		Sensitivity	100.0%
		Specificity	100.0%
		Accuracy	100.0%
(b)			
Within mandatory DVH tolerances	10 (71.4%)	True negative	9 (90.0%)
		False negative	1 (10.0%)
Exceeding mandatory DVH tolerances	4 (28.6%)	True positive	2 (50.0%)
		False positive	2 (50.0%)
		Sensitivity	50.0%
		Specificity	90.0%
		Accuracy	78.6%

*Note*: Four manual treatment plans exceed mandatory tolerances due to tumor control probability (TCP) prioritization over normal tissue complication probability (NTCP) as described previously.

Network sensitivity to out‐of‐tolerance plans was 1.0 when considering optimal treatment planning constraints and 0.5 for mandatory constraints.

### 3.4 Risk‐based RS stratification

**FIGURE 6:**
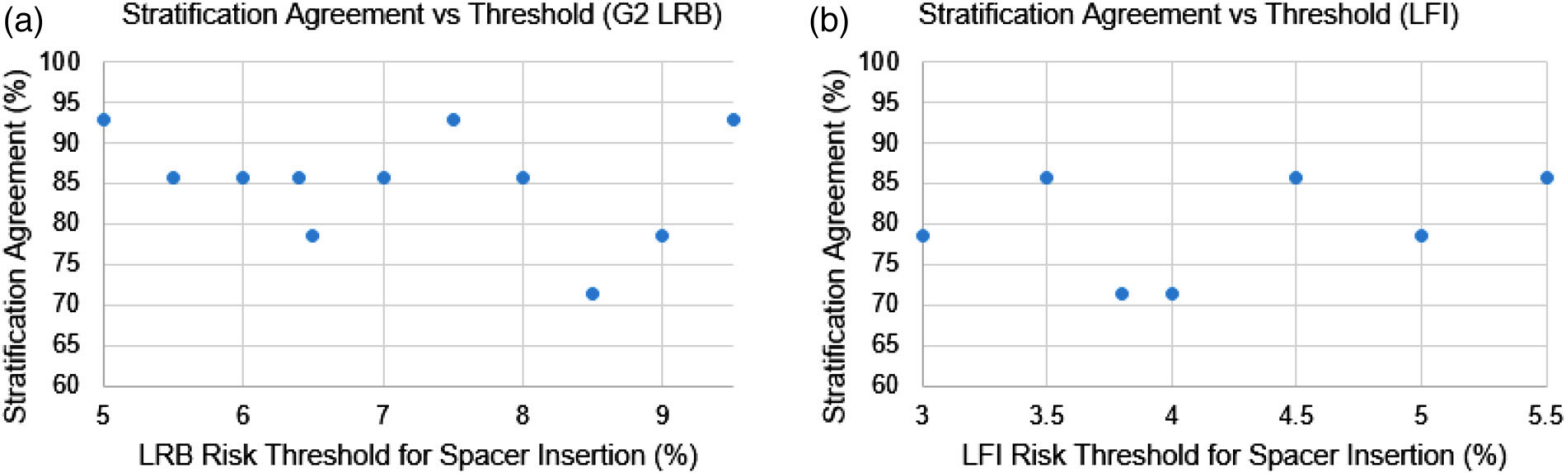
Agreement between neural‐network (NN) predicted and manually planned risk stratification as a function of risk threshold for (a) late rectal bleeding (LRB) and (b) late fecal incontinence (LFI)

The accuracy of the network to stratify patients based on predicted toxicity risk was above 71% for LRB for all thresholds (Figure 6a). The median G2 LRB risk for the test cohort was 6.4%. If a center could afford to offer RS insertion to half of their patients with risk ≥6.4%, 86% of patients would be correctly stratified. A similar trend was seen for stratification based on predicted LFI risk, with 71% correctly stratified around the cohort median LFI risk of 3.8% (Figure 6b).

## 4 DISCUSSION

RS insertion reduces rectal dose in patients treated with RT to the prostate, however where resources are not routinely available for spacer insertion, centers must make decisions on an individual patient basis. Much of the patient‐ and resource‐related factors^3^ are known at the time of decision‐making, apart from radiation dose to the rectum which may take days to estimate using standard RT planning. This study demonstrates a method for rapidly predicting rectal dose and rectal toxicity directly after target and OAR delineation, with sufficient accuracy to assist in decision‐making. There has been recent interest in designing NN dose prediction models to assist the treatment planning process,^8^, ^9^, ^10^, ^11^, ^12^, ^13^ with models that act as direct decision‐support systems the next logical step.

PTV dose prediction was highly accurate, which is to be expected as the manual treatment planning process stipulates homogeneous dose to each PTV, yielding a simple relationship between PTV geometry and PTV dose distribution. Prediction of dose outside the PTVs is more complex as patient anatomies differ, and the manually interactive treatment planning process gives rise to variations in dose fall‐off and dose distribution between patients and plans.^20^

In terms of rectal DVH prediction, a small and clinically negligible bias was seen for each DVH parameter in the validation cohort, with larger positive bias seen in the test cohort (Figure 3). This discrepancy can be partially attributed to a bias in the training dataset to posterior boost regions. The clinical test dataset contained some boost regions further from the rectum for which the network was unfamiliar and consequently over‐predicted rectal dose. For both cohorts better network performance is seen at the upper and lower extents of the DVH. The lower doses were mostly in the regions superior and inferior to the PTVs which tend to be more similar between patients, determined by the craniocaudal rectal length relative to PTV length, and the way in which the TPS models scattered dose from the primary beam. The network also performed better in the dose region above 53 Gy on account of restrictive treatment planning constraints. The largest uncertainties occurred in the mid‐range doses, where dose is primarily dictated by the axial dose fall‐off from PTV53 through the rectum. This dose gradient is directly influenced by patient anatomy and by the operator‐guided inverse optimization procedure within the treatment planning process, resulting in more variation.

It is difficult to make comparisons with the existing dose prediction literature due to differing patient cohorts, treatment modalities, clinical protocols, and analysis, but the closest example is Nguyen et al.^11^ who reported a dose prediction network for single dose level prostate plans. The overall mean absolute errors were 1.8% ± 1.1% (1 SD) and 1.0% ± 0.6% for PTV Dmax and Dmean, respectively, which compares favorably with our results of 1.2% ± 1.0% and 0.2% ± 0.2% for the primary target (PTV60) Dmax and Dmean. Likewise, prediction of rectal Dmax had similar mean absolute errors of 1.6% ± 1.1%^11^ compared with 1.4% ± 0.9% in the current study. Absolute errors in rectal Dmean prediction were however greater in our study at 3.9% ± 2.8% compared with 1.6% ± 1.1% in the published work. Reasons for this may include our network being tasked with predicting the more complex dose fall‐off from a three dose level plan rather than a single prescription level plan. In addition, our study was aimed at predicting dose distributions from volumetric modulated arc therapy (VMAT) plans with more degrees of freedom than the standardized seven‐field intensity modulated RT (IMRT) plans used in the work by Nguyen et al. However, the main reason for the larger errors we observed is likely to be the smaller size of our training dataset, which comprised 30 patients with six augmented plans compared to the 72 patients in Nguyen et al., as larger training datasets make for a more robust and accurate network. While mean dose is a good indicator of general DVH agreement, it should be noted that the toxicity models used within this work (especially LRB) are reliant on accurate prediction of mid to higher rectal doses, and therefore evaluation of mean dose, which applies the same level of weight to all parts of the rectal DVH, may be less clinically relevant.

Prediction of 3D dose distribution in the vicinity of the rectum is important as it provides qualitative validation of the network's numerical DVH prediction results and a visual representation of the high doses delivered to the rectum, which may indicate to the clinical care team and patient any need for RS implantation. Our results gave consistently high values for 3D dose prediction over the sampled dose range, with average DSC of 0.94 — a similar accuracy to the NN‐derived dose predictions of Nguyen et al.^11^ (mean DSC of 0.91). In many cases the predicted isodoses in the vicinity of the rectum were close to manually planned isodoses, as shown in Figure 4a. Figure 4 also illustrates isodoses for two of the plans with lowest prediction accuracy. Local isodose disagreement is witnessed inside the rectum, with higher accuracy around the PTVs. It is encouraging to see the predicted isodose lines affected by the presence of the rectum, forming visibly concave isodoses, but for these extreme cases rectal dose is either under‐ or over‐predicted. Several factors may be involved. Firstly, the small training dataset likely restricts network performance, meaning the network does not generalize well to some unseen patient anatomies. The second factor may lie in the inherent variability in the manual planning process.

Prediction of toxicity risk was good for G2 LRB as the LKB parameters focus on mid‐ to high‐dose range within the DVH where the network performs well. Despite the larger uncertainties in DVH prediction at the low‐ to mid‐dose range, the 95% accuracy of predicting LFI risk was better than LKB, albeit with a positive bias.

To evaluate the results further and put them into clinical context, it is noteworthy that the process of manual planning is an iterative, trial and error approach where the planner navigates to a solution in the time allowed, leading to variation in plan quality.^20^ There is inherent variability in manually planned rectal DVHs^21^ and subsequent rectal toxicity risk for RT to the prostate.^22^ Scaggion et al.^21^ reported interquartile ranges (IQRs) for rectal V30Gy, V40Gy, V50Gy, V60Gy, V65Gy, V70Gy, and V75Gy of 17.37%, 11.69%, 6.70%, 4.26%, 3.51%, 2.79%, and 0.81%, respectively. Our network performed with similar or better accuracy, with IQRs of 9.03%, 9.61%, 6.47%, 4.35%, 3.31%, 1.88%, and 1.24%, respectively (doses equated by linear quadratic conversion to equivalent dose in 2 Gy fractions, with *α*/*β* = 3 Gy). Moore et al.^22^ assessed the increased late rectal toxicity risk introduced by suboptimal manual planning quoting increases of up to 17%, with a mean excess risk of 4.7% (±3.9% SD). For LRB, our dose prediction network had limits of agreement which are small compared to the amount of variation in risk prediction arising from manual planning.

The similarity between target and rectal volumes in the study cohort and clinically treated patients provides some assurance that the network is likely to be sufficiently robust for clinical application at our center. Also in relation to the clinically treated cohort of 100 patients at our center, the network performed well within the observed variation of toxicity risk estimations.

Several authors have performed cost–benefit analyses for RS insertion^2^ but there is an awareness that the cost‐effectiveness for healthcare providers can be increased through appropriate selection of suitable patients.^3^ The network performed well when stratifying patients for RS insertion based on prediction of out‐of‐tolerance DVH planning constraints, with good sensitivity to out‐of‐tolerance treatment plans, and acceptable prediction accuracy.

The current model predicts patient toxicity risk with 95% confidence limits in the region of 1.5%; the same order of magnitude as observed toxicity risk ranges of around 5% (Figure 5). When evaluating the significance of this in terms of risk‐based RS stratification, this level of accuracy results in good stratification performance for both LRB and LFI with accuracy over 71% irrespective of threshold used. To put this into practice, centers would need to perform a cost–benefit evaluation whereby a suitable threshold is based on their own patient population, and available resources. While it may be advisable to manually plan those plans where network predicted toxicity risk is close to the tolerance level (to confirm stratification result), the network performance allows a considerable amount of manual planning to be avoided, thus saving time and valuable planning resources.

While the dose prediction itself takes less than a second this must be incorporated within a workflow to extract anatomical data from the TPS, perform the NN dose prediction, estimate toxicity, and stratify the patient as high or low risk. With suitable hardware available to the clinician, this whole process takes less than 5 min and can be carried out directly after delineation of the required targets and healthy tissue. This amounts to a significant reduction in time over conventional treatment planning and can be actioned immediately rather than being subject to clinical pathway and workload pressures. When combined into a pipeline with artificial intelligence (AI)‐based auto‐segmentation, a decision on RS stratification could be established directly after imaging the patient. In addition, such rapid prediction of dose and toxicity could be applied to simulated “virtual spacers”^23^ to predict the magnitude of rectal toxicity reduction and the associated cost–benefit of spacer insertion.

This study has some limitations. The treatment planning was performed by only one operator. This optimal situation allows the model to be trained on consistently planned data subject only to intra‐operator variation in plan quality, with no inter‐operator variation. In a clinical situation however, this ideal may not be realized due to clinical resource restrictions. It is also acknowledged that as the DILs were simulated within the training cohort, their position and size will differ from clinically derived lesions despite our process of matching DIL volumes to data reported in the literature. This is seen to some extent by the positive bias when moving to our test cohort (Figure 3), and future work will involve simulating boost regions in the training dataset further from the rectum. The training dataset had few cases compared to other published dose prediction networks^8^, ^9^, ^12^, ^13^ which have used between 72 and 195 training datasets, nonetheless accuracy was encouraging. Further training datasets will likely increase the rectal dose prediction accuracy.

It is acknowledged that should the patient be stratified for RS, a repeat RT planning CT scan would be required post‐insertion, which incurs additional cost and imaging dose for the patient. Further benefits in terms of patient pathway, utilization of staff resources, and patient imaging dose could therefore be realized by predicting rectal dose and toxicity from pre‐existing diagnostic imaging rather than the RT planning CT scan. However, diagnostic imaging is performed on curved couches, with the patient in non‐RT position, and is typically performed prior to months of androgen deprivation therapy which can significantly alter the volume of the prostate. For the time‐being, the decision‐support tool is most accurately used at the point of RT planning as described in this study.

## 5 CONCLUSIONS

This study proposes a dose prediction NN as a resource‐efficient decision‐support system for stratifying patients at high risk of toxicities for surgical insertion of RS prior to RT and is, to our knowledge, the first to do so in the literature. In the arena of highly complex, dose‐escalated, toxicity‐guided prostate RT the network predicted rectal dose distributions in only 0.7 s with an encouraging level of accuracy, correctly stratifying over 86% of patients for the procedure and identifying those patients close to tolerance where standard treatment planning would be required. While not yet ready for clinical implementation, the accuracy of toxicity risk prediction translates into an encouraging level of stratification accuracy using our model. As such, this work provides proof‐of‐principle that a real‐time dose prediction model can be used in a novel way to support rapid decision‐making when stratifying patients for an intervention, and thus can play an important role in improving value of care through better utilization of resources. However, further network training followed by a more expansive clinical implementation study on a larger number of patient datasets is needed prior to clinical deployment.

## CLINICAL TRIAL INFORMATION

Patient data are taken from an ethically approved (UK Health Research Authority) clinical trial running at Guy's and St. Thomas’ NHS Foundation Trust.

## CONFLICT OF INTEREST

The authors have no relevant conflicts of interest to disclose.

## Supporting information

SUPPORTING INFORMATION

## Data Availability

Authors are not able to share data at this time.

## References

[1] Cancer Research UK . Prostate Cancer Statistics . Accessed August 16, 2021. https://www.cancerresearchuk.org/health‐professional/cancer‐statistics/statistics‐by‐cancer‐type/prostate‐cancer

[2] National Institute for Health and Care Excellence . Biodegradable spacer insertion to reduce rectal spacer insertion to reduce rectal toxicity during radiotherapy for prostate cancer. *Interventional Procedures Guidance [IPG590]*. Accessed August 16, 2021. https://www.nice.org.uk/guidance/ipg590

[3] Vanneste BGL , Hoffmann AL , van Lin EN , Van De Voorde L , Pinkawa M , Lambin P . Who will benefit most from hydrogel rectum spacer implantation in prostate cancer radiotherapy? A model‐based approach for patient selection. Radiother Oncol. 2016;121(1):118‐123. 10.1016/j.radonc.2016.08.026 27647458

[4] Michalski JM , Gay H , Jackson A , Tucker SL , Deasy JO . Radiation dose‐volume effects in radiation‐induced rectal injury. Int J Radiat Oncol Biol Phys. 2010;76(3 suppl):123‐129. 10.1016/j.ijrobp.2009.03.078 20171506 PMC3319467

[5] Lips IM , van der Heide UA , Haustermans K , et al. Single blind randomized phase III trial to investigate the benefit of a focal lesion ablative microboost in prostate cancer (FLAME‐trial): study protocol for a randomized controlled trial. Trials. 2011;12(1):255. 10.1186/1745-6215-12-255 22141598 PMC3286435

[6] Monninkhof EM , van Loon JWL , van Vulpen M , et al. Standard whole prostate gland radiotherapy with and without lesion boost in prostate cancer: toxicity in the FLAME randomized controlled trial. Radiother Oncol. 2018;127(1):74‐80. 10.1016/j.radonc.2017.12.022 29336835

[7] Syndikus I , Cruickshank C , Staffurth J , et al. PIVOTALboost: a phase III randomised controlled trial of prostate and pelvis versus prostate alone radiotherapy with or without prostate boost (CRUK/16/018). Clin Transl Radiat Oncol. 2020;25:22‐28. 10.1016/j.ctro.2020.08.003 32995575 PMC7508714

[8] Nguyen D , Jia X , Sher D , et al. 3D radiotherapy dose prediction on head and neck cancer patients with a hierarchically densely connected U‐net deep learning architecture. Phys Med Biol. 2019;64(6):065020. 10.1088/1361-6560/ab039b 30703760

[9] Mardani M , Dong P , Xing L . Deep‐learning based prediction of achievable dose for personalizing inverse treatment planning. Int J Radiat Oncol. 2016;96(2):E419‐E420. 10.1016/j.ijrobp.2016.06.1685

[10] Shiraishi S , Moore KL . Knowledge‐based prediction of three‐dimensional dose distributions for external beam radiotherapy. *Med Phys*. 2016;378(1):378‐387. 10.1118/1.4938583 26745931

[11] Nguyen D , Long T , Jia X , et al. A feasibility study for predicting optimal radiation therapy dose distributions of prostate cancer patients from patient anatomy using deep learning. Sci Rep. 2019;9(1):1‐10. 10.1038/s41598-018-37741-x 30705354 PMC6355802

[12] Kearney V , Chan JW , Haaf S , Descovich M , Solberg TD . DoseNet: a volumetric dose prediction algorithm using 3D fully‐convolutional neural networks. Phys Med Biol. 2018;63. 10.1088/1361-6560/aaef74 30511663

[13] Fan J , Wang J , Chen Z , Hu C , Zhang Z , Hu W . Automatic treatment planning based on three‐dimensional dose distribution predicted from deep learning technique. Med Phys. 2019;46(1):370‐381. 10.1002/mp.13271 30383300

[14] Onjukka E , Uzan J , Baker C , Howard L , Nahum A , Syndikus I . Twenty fraction prostate radiotherapy with intra‐prostatic boost: results of a pilot study. Clin Oncol. 2017;29(1):6‐14. 10.1016/j.clon.2016.09.009 27692920

[15] Chen ME , Johnston DA , Tang K , Joseph Babaian R , Troncoso P . Detailed mapping of prostate carcinoma foci: biopsy strategy implications. Cancer. 2000;89(8):1800‐1809. 10.1002/1097-0142(20001015)89:8<1800::aid‐cncr21>3.0.co;2‐d 11042576

[16] McNeal JE , Redwine EA , Freiha FS , Stamey TA . Zonal distribution of prostatic adenocarcinoma. Correlation with histologic pattern and direction of spread. Am J Surg Pathol. 1988;12(12):897‐906. 10.1097/00000478-198812000-00001 3202246

[17] Nutting CM , Corbishley CM , Sanchez‐Nieto B , Cosgrove VP , Webb S , Dearnaley DP . Potential improvements in the therapeutic ratio of prostate cancer irradiation: dose escalation of pathologically identified tumour nodules using intensity modulated radiotherapy. Br J Radiol. 2002;75(890):151‐161. 10.1259/bjr.75.890.750151 11893639

[18] Bauman G , Haider M , Van Der Heide UA , Ménard C . Boosting imaging defined dominant prostatic tumors: a systematic review. Radiother Oncol. 2013;107(3):274‐281. 10.1016/j.radonc.2013.04.027 23791306

[19] Uzan J , EswarVee C , Malik Z , Nahum AE . Biosuite, new software for radiobiological customisation of dose and fraction size in EBRT. Radiother Oncol. 2009;92(suppl 1):S239. 10.1016/S0167-8140(12)73231-0

[20] Nelms BE , Robinson G , Markham J , et al. Variation in external beam treatment plan quality: an inter‐institutional study of planners and planning systems. Pract Radiat Oncol. 2012;2(4):296‐305. 10.1016/j.prro.2011.11.012 24674168

[21] Scaggion A , Fusella M , Roggio A , et al. Reducing inter‐ and intra‐planner variability in radiotherapy plan output with a commercial knowledge‐based planning solution. Phys Med. 2018;53:86‐93. 10.1016/j.ejmp.2018.08.016 30241759

[22] Moore KL , Schmidt R , Moiseenko V , et al. Quantifying unnecessary normal tissue complication risks due to suboptimal planning: a secondary study of RTOG 0126. Int J Radiat Oncol Biol Phys. 2015;92(2):228‐235. 10.1016/j.ijrobp.2015.01.046 25847605 PMC4431941

[23] van Wijk Y , Vanneste BGL , Walsh S , et al. Development of a virtual spacer to support the decision for the placement of an implantable rectum spacer for prostate cancer radiotherapy: comparison of dose, toxicity and cost‐effectiveness. Radiother Oncol. 2017;125(1):107‐112. 10.1016/j.radonc.2017.07.026 28823404

