# Supplementary material for: Neural network dose prediction for rectal spacer stratification in dose‐escalated prostate radiotherapy
Source: Med Phys. 2022 Mar 11;49(4):2172–82. doi: 10.1002/mp.15575 (PMC9311720; doi:10.1002/mp.15575)
Supplement: Supplementary file 1 — SUPPORTING INFORMATION [file MP-49-2172-s001.pdf]

Supplementary Material

a)

Optimal Tol.	Manually - planned Dose Exceeds	Network-Predicted Dose Exceeds
V24.6Gy < 70%	19 (15.8%)	24 (20%)
V32.4Gy < 60%	9 (7.5%)	12 (10%)
V40.8Gy < 50%	4 (3.3%)	6 (5%)
V48.6Gy < 30%	0 (0%)	6 (5%)
V52.8Gy < 15%	0 (0%)	0 (0%)
V57Gy < 3%	0 (0%)	0 (0%)
V60Gy < 1%	60 (50%)	64 (53.3%)
V64Gy < 0.2%	88 (73.3%)	93 (77.5%)
V68Gy < 0.2%	2 (1.7%)	0 (0%)

b)

Mandatory Tol.	Manually-planned Dose Exceeds	Network-Predicted Dose Exceeds
-	- -	- -
-	- -	- -
V40.8Gy < 60%	0 (0%)	6 (5%)
V48.6Gy < 50%	0 (0%)	0 (0%)
V52.8Gy < 30%	0 (0%)	0 (0%)
V57Gy < 15%	0 (0%)	0 (0%)
V60Gy < 5%	28 (23.3%)	28 (23.3%)
V64Gy < 1%	32 (26.7%)	27 (22.5%)
V68Gy < 0.2%	2 (1.7%)	0 (0%)

Table S-1: Number of a) optimal and b) mandatory rectal DVH objectives exceeded for manually-planned and network-predicted dose distributions in the validation cohort of 120 plans

a)

Optimal Tol.	Manually - planned Dose Exceeds	Network-Predicted Dose Exceeds
V24.6Gy < 70%	0 (0%)	0 (0%)
V32.4Gy < 60%	0 (0%)	0 (0%)
V40.8Gy < 50%	0 (0%)	0 (0%)
V48.6Gy < 30%	0 (0%)	0 (0%)
V52.8Gy < 15%	0 (0%)	0 (0%)
V57Gy < 3%	0 (0%)	0 (0%)
V60Gy < 1%	6 (42.9%)	7 (50%)
V64Gy < 0.2%	9 (64.3%)	9 (64.3%)
V68Gy < 0.2%	0 (0%)	0 (0%)

b)

Mandatory Tol.	Manually-planned Dose Exceeds	Network-Predicted Dose Exceeds
-	-	-
-	-	-
V40.8Gy < 60%	0 (0%)	0 (0%)
V48.6Gy < 50%	0 (0%)	0 (0%)
V52.8Gy < 30%	0 (0%)	0 (0%)
V57Gy < 15%	0 (0%)	0 (0%)
V60Gy < 5%	4 (28.6%)	3 (21.4%)
V64Gy < 1%	3 (21.4%)	2 (14.3%)
V68Gy < 0.2%	0 (0%)	0 (0%)

Table S-2: Number of a) optimal and b) mandatory rectal DVH objectives exceeded for manually-planned and network-predicted dose distributions in the test cohort of 14 plans.

a)

	Manual Plans	Network Prediction	
Within optimal tol.	21 (17.5%)	true -ve	14 (66.7%)
		false -ve	7 (33.3%)
Exceeding optimal tol.	99 (82.5%)	true +ve	97 (98%)
		false +ve	2 (2%)
		Sensitivity	98.0%
		Specificity	66.7%
		Stratification Accuracy	92.5%

b)

	Manual Plans	Network Prediction	
Within mandatory tol.	86 (71.7%)	true -ve	80 (93%)
		false -ve	6 (7%)
Exceeding mandatory tol.	34 (28.3%)	true +ve	29 (85.3%)
		false +ve	5 (14.7%)
		Sensitivity	85.3%
		Specificity	93.0%
		Stratification Accuracy	90.8%

Table S-3: Validation cohort. Number of manual treatment plans and network-predicted treatment plans within or exceeding a) optimal and b) mandatory rectal DVH constraints. 34 out of 120 manual treatment plans exceed mandatory tolerances due to TCP prioritisation over NTCP as described previously.

	Manually-Planned	Predicted	Predicted – Truth
	(mean ± stdev)	(mean ± stdev)	(mean ± stdev)
PTV68 D50% (Gy)	68.1 ± 0.1	68.3 ± 0.2	0.3 ± 0.3
PTV60 D50% (Gy)	62.7 ± 0.2	62.7 ± 0.3	0.0 ± 0.2
PTV53 D50% (Gy)	59.7 ± 1.0	59.6 ± 1.0	-0.1 ± 0.1

Table S-4: PTV median dose statistics over all patients.

	Average Absolute Dose Difference (%) mean value ± 1 standard deviation	
	Dmax	Dmean
PTV68	0.75 ± 0.45	0.53 ± 0.38
PTV60*	1.22 ± 1.04	0.22 ± 0.16
PTV53*	2.04 ± 1.57	0.36 ± 0.27
Rectum	1.42 ± 0.87	3.91 ± 2.79

Table S-5: Average absolute dose difference statistics in test cohort, as a percentage of 60Gy prescription dose. *Higher dose PTVs excluded from structure

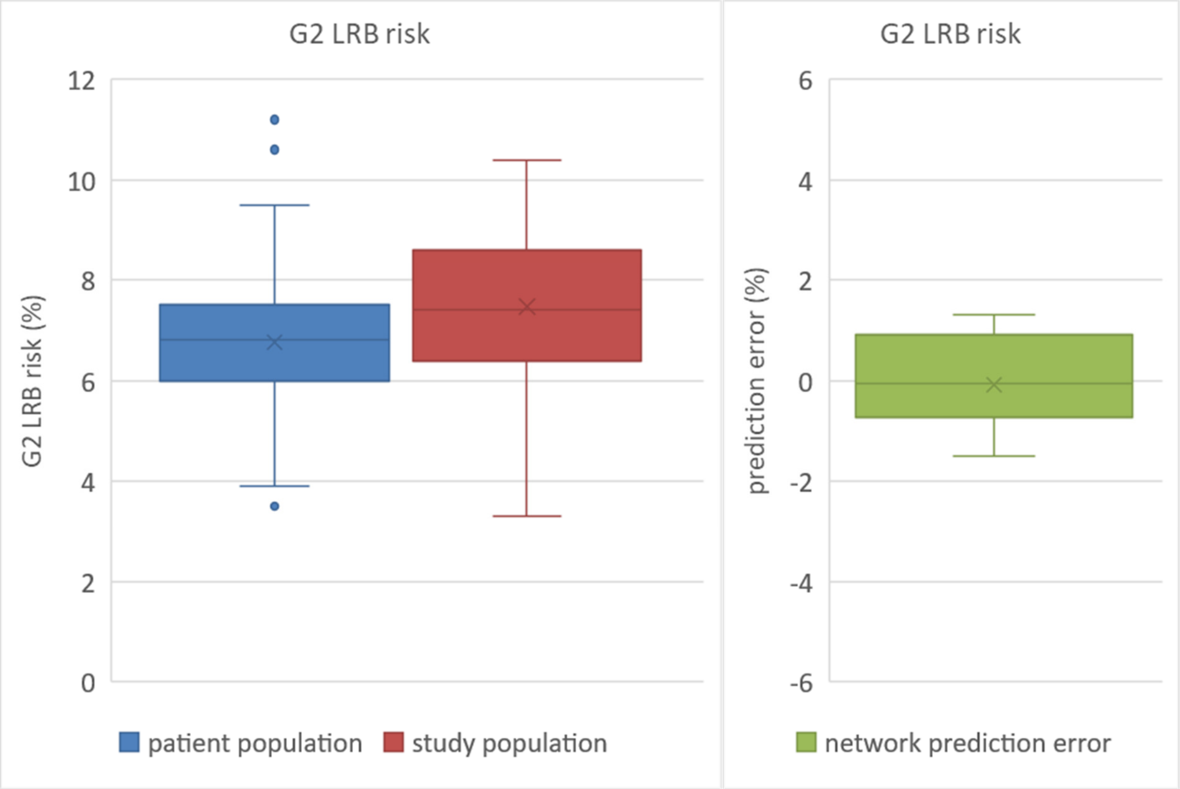

Figure S-1: G2 LRB risk for clinical and study cohort, with NN risk prediction error for comparison

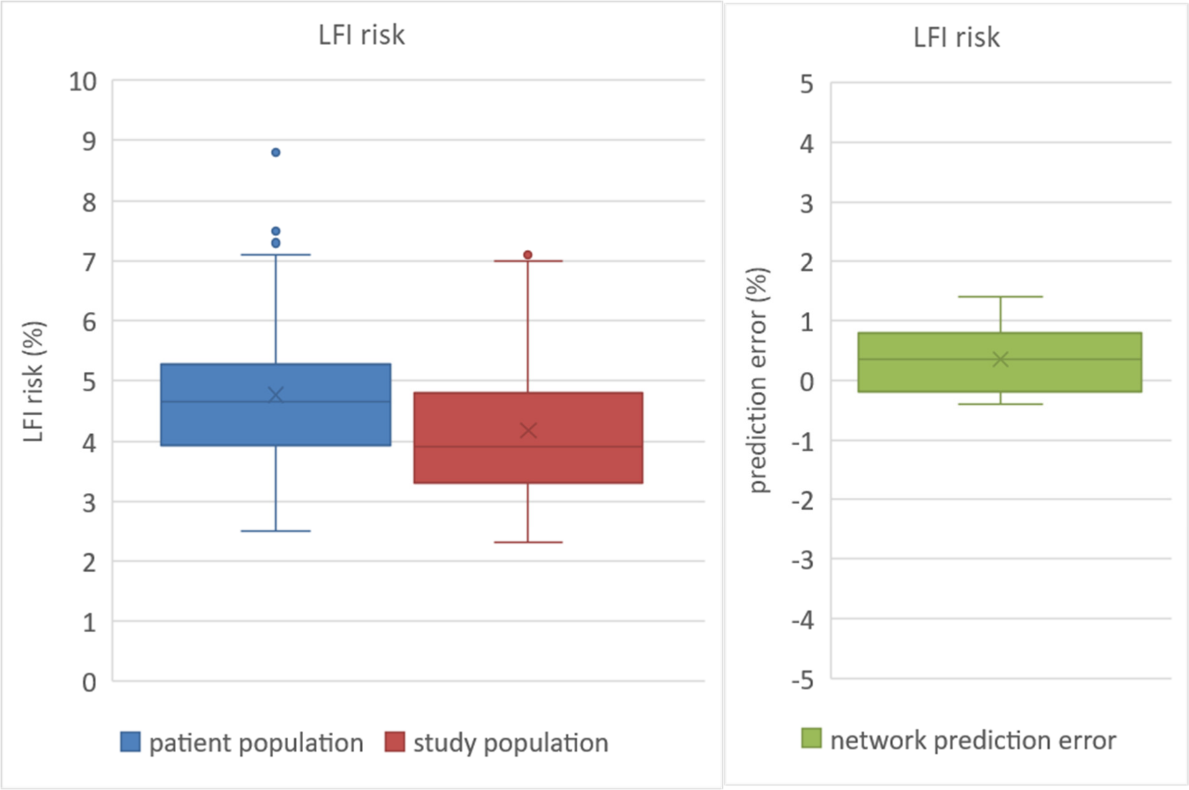

Figure S-2: G2 LFI risk for clinical and study cohort, with NN risk prediction error for comparison

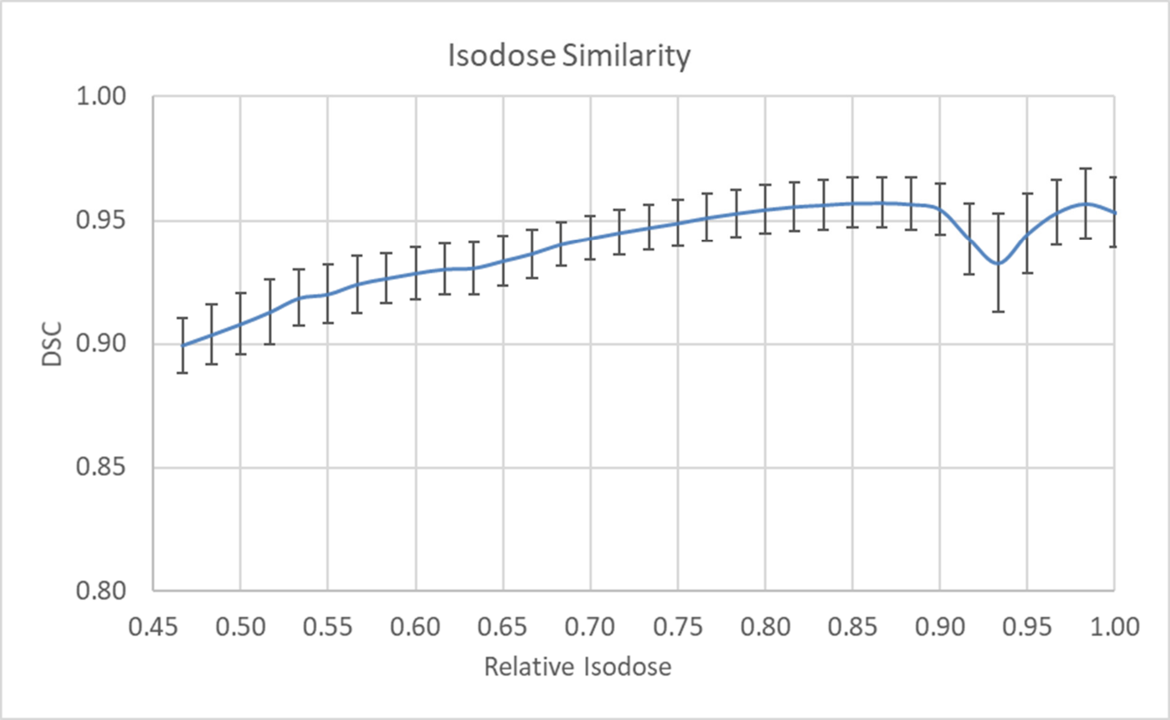

Figure S-3: Dice similarity coefficients (DSC) for test cohort, comparing predicted isodoses against manually-planned isodoses, averaged over all plans. Error bars indicate 1 standard deviation.
